# On Soldering in Dentistry

**Published:** 1882-12

**Authors:** W. H. Dorrance

**Affiliations:** Professor of Prosthetic Dentistry and Dental Metallurgy, in the Dental College of the University of Michigan


### On Soldering in Dentistry.

[W. H. Dorrance, D. D. S., Professor of Prosthetic Dentistry and Dental Metallurgy, in the Dental College of the University of Michigan.]

Prominent among the difficulties of manipulation which combine to retard the return to the use of the metals in prosthetic dentistry, is that of soldering; and after the principles involved in the preparation of the piece, investment and proper application of heat are mastered, without a good solder—which is the objective point of this article—the result is a failure to a greater or less degree.

In the consideration of some of the points involved, two general principles should first be noted, viz; (1) the *ne plus ultra* of hard-soldering is the partial fusion of the contact surface at the moment of perfect fusion of the solder; therefore, (2) the nearer the solder in its melting point and composition is to the metals or alloys to be united, and the more nearly perfect the contact of the parts, the stronger and more perfect the joint. An illustration of (1) being the use of pure gold in soldering platinum for continuous gum work, as, when the piece is fixed, the base is so near the fusing point, and the gold so reduces it by its presence, that union of the platinum with itself takes place.

The choice of the means of uniting two or more pieces of metal by the method called hard-soldering, involves some or all of the following points: (*a*) the fusing point of the metals or alloys to be united; (*b*) the uses to which the soldered piece is to be put; (*c*) the mechanical or chemical action to which it is subject; (*d*) the individual action of the metals used to render the solder fusible upon the metals to be united, and (*e*) the color. Considering them in their order, note the following facts.

(*a*.) Platinum fuses at about 3992° F., the only solder admissible being pure gold, as the attempt to use alloyed gold (solder) or silver solder results in a very weak joint, with the tendency to destruction of the integrity of the platinum. Pure gold is usually said to fuse at 2016° F., but 2282° F. is nearer the mark. The addition of the usual alloys, copper (fusing at about 1996°), or silver (fusing at about 1873°), or both, lowers the fusing point of the gold (alloy) so that a solder must contain a still larger proportion of these metals, and even the addition of a metal fusing at a much lower temperature, preferably zinc (fusing at 773°). Pure silver fuses at about 1873° F. The addition of copper, its proper alloy, lowers its fusing point, and solder must contain still more copper, and eventually the addition of zinc, as in the case of the solder for gold. Arsenic (metallic) is, for some purposes, also added to silver solder, making it whiter and more fusible, but brittle.

(*b.*) The support of an artificial denture of greater or less extent need only be considered in this connection, and for this purpose.

(*c.*) The strain to which the individual teeth and the plate are subjected in the act of mastication call for strength of solder; and the solvent action of the secretions when vitiated, and of acids introduced in the food and as medicines, with the possibility of induced galvanic action, calls for as near an approach to purity in the solder as possible. The addition of zinc to the solder, except in quite small quantities, renders it particularly susceptible to these influences.

(*d.*) Platinum is rapidly affected by zinc and other base metals. Gold is rendered exceedingly brittle by presence of a minute quantity of arsenic, cadmium, tin, lead or antimony. Silver is also more or less affected by the above base metals. Commercial zinc is usually contaminated with arsenic, antimony, etc., and is therefore unfit to use as an alloy in solder for the dentists' use, but on the contrary pure zinc may be used in small proportions with good effect, causing gold and silver solders to be more fusible and to flow more readily. If the addition of it is carried too far, however, it causes the solder to "eat" into the metal or alloy on which it is used. Copper is also liable to be contaminated, and the pure metal alone should be used in solder and other alloys used in the mouth.

(*e.*) As in other work of art, so in an artificial denture, there should be the least possible difference in the color of the plate and the solder, which should, in other words, follow the color of the plate used.

The following are usual sources of trouble to the unpracticed worker: (1) the solder is not of the supposed quality, the proportions of the metals entering into the alloy having changed by being carelessly fused; or, (1), it may be made after a poor formula. (2) The surfaces to be soldered and of the solder, are not sufficiently clean, or in contact. (3) Too little or too much flux is used. (4) The case is improperly heated and the solder fused (and kept so) long before the case is ready for it.

In order, then, to accomplish the best results in soldering a piece which is to be worn in the mouth, (1) the solder, whatever its degree of fineness, must be (*a*) as near the character and quality of the plate as possible and flow well; (*b*) care must be taken in making it that the proportions of the metal entering into its composition be not changed; (*c*) it must be in a form to be readily handled, and (*d*) care must be taken as to the amount used. Now, (*a*), platinum, as before stated, should be soldered only with pure gold; pure gold may be soldered with, say, coin gold; coin gold (900-1000 fine) with, say, 20k. solder; 20k. gold-plate (say, 835-1000 fine), with, say, not less than 18k. solder. The addition of from 5 to 8 per cent. of cadmium (as suggested to the writer at the last session of the A. D. A.), with a fusing point of 442° F., to pure gold or gold plate to form a solder, causes it to flow very easily, and, with care in the management of the flame, the cadmium is to a great extent volatilized, but sufficient invariably remains to render the gold more brittle than with a pure solder. (*b*) In making solder even more pains should be taken than in the forming of other common alloys, the same general rules being followed; the metals of about the same fusing point being first melted, and then those of a lower fusing point carefully added; the perfect protection of the surface during fusion, usually with borax, but with powdered charcoal where zinc is added and it is wished to preserve the proportion of it; and it is essential that fusion be kept up no longer than necessary to the complete mixing of the alloy. (*c*) A good rule in regard to the form of the solder is to roll it into plate of about, or a little more than the thickness of the plate to be soldered, anneal it, and thoroughly cleanse the surface. (*d*) Inasmuch as the perfect joint is one where the parts to be united are in contact, and there is fusion of the contact surfaces, it follows that the amount of solder used should be just sufficient to fill the joint, except where it is desirable to give form or contour; and this involves the use of as fine solder as possible. (1*a*) Good or poor formulas will be hereafter referred to.

(2) It should be borne in mind that solder will not flow over an oxydized surface, neither will it fill a hole (unless quite small), nor a wide joint, though it perfectly follows the law of capillary attraction; so it is essential that the surfaces to be united or covered with the solder be bright and clean, and the parts in contact.

(3) Good judgment is needed in the application of the flux, the surface is not sufficiently protected where too little is used, and the surface and solder are oxydized, and too much not only prevents the flow of the solder, but by its bulk causes the solving quality of the fused borax to be too much for the lower grade metals forming the alloy. The borax should be ground with clean water on a hard, rough-grained, slate (soft slate mixes with the borax in such quantity as to be in the way, as it is not fused), or a rough surfaced porcelain slab, to the consistency of cream—no thinner or thicker—and painted on the solder or surfaces to be soldered, and even the whole exposed surface, if it is desirable to protect it from oxydation; or the calcined and powdered borax may be ground with water to the same consistence and used in the same way; or, in a delicate case of soldering, the cold saturated solution of borax may be used to a good advantage. This does not hinder the application of a little borax in the lump to the case during the soldering process, should it be required.

(4) With the most careful preparation and the best of solder, the case may be, and not infrequently is, ruined by the improper application of heat. The case, whether involving investment or not, should be gradually and evenly brought quite up to the fusing point of the solder (preferably, if an invested case, in the charcoal furnace), without allowing an excess of heat to reach it, or any delicate part to become unduly heated, and then, with a small flame, the solder may be easily drawn where it is most desired. Should any other course be pursued, such, for instance, as the rapid application of heat to that part alone that is to be soldered, teeth are checked, if present, the case is sprung from unequal expansion, delicate parts or edges, backings or rims are fused, and the solder is ruined and made worse than useless by the loss of its more volatile constituents, and the presence of the oxides formed.

